# Supplementary material for: Influence of health literacy on acceptance of influenza and pertussis vaccinations: a cross-sectional study among Spanish pregnant women
Source: BMJ Open. 2018 Jul 6;8(7):e022132. doi: 10.1136/bmjopen-2018-022132 (PMC6042548; doi:10.1136/bmjopen-2018-022132)
Supplement: Author's manuscript [file bmjopen-2018-022132.draft_revisions.pdf]

BMJ Open is committed to open peer review. As part of this commitment we make the peer review history of every article we publish publicly available.

When an article is published we post the peer reviewers' comments and the authors' responses online. We also post the versions of the paper that were used during peer review. These are the versions that the peer review comments apply to.

The versions of the paper that follow are the versions that were submitted during the peer review process. They are not the versions of record or the final published versions. They should not be cited or distributed as the published version of this manuscript.

BMJ Open is an open access journal and the full, final, typeset and author-corrected version of record of the manuscript is available on our site with no access controls, subscription charges or pay-per-view fees (http://bmjopen.bmj.com).

If you have any questions on BMJ Open's open peer review process please email info.bmjopen@bmj.com

BMJ Open

Influence of health literacy on acceptance of influenza and pertussis vaccinations among Spanish pregnant women

Journal:	*BMJ Open*
Manuscript ID	bmjopen-2018-022132
Article Type:	Research
Date Submitted by the Author:	02-Feb-2018

Complete List of Authors:	Castro-Sánchez, Enrique; Imperial College London,<br>Vila-Candel, Rafael; Hospital Universitario de la Ribera, Department of Obstetrics and Gynaecology,; Universidad Católica de Valencia 'San Vicente Mártir', Faculty of Nursing<br>Soriano-Vidal, Francisco; Foundation for the Promotion of Health and Biomedical Research in the Valencian Region (FISABIO)., Xàtiva-Ontinyent Health Department; Universidad Católica de Valencia 'San Vicente Mártir', Faculty of Nursing<br>Navarro-Illana, Esther; Universidad Católica de Valencia 'San Vicente Mártir', Faculty of Nursing<br>Domingo, Javier Diez; Foundation for the Promotion of Health and Biomedical Research in the Valencian Region (FISABIO).; Universidad Católica de Valencia 'San Vicente Mártir', Faculty of Nursing

Keywords:	Immunisation, Influenza, Pertussis, Health literacy, Maternal medicine < OBSTETRICS

SCHOLARONE™
Manuscripts

INFLUENCE OF HEALTH LITERACY ON ACCEPTANCE OF INFLUENZA AND PERTUSSIS VACCINATIONS AMONG SPANISH PREGNANT WOMEN

Authors

Enrique Castro-Sánchez[1], Rafael Vila-Candel[2,3], Francisco J Soriano-Vidal[3,4,5,6], Esther Navarro-Illana[3], Javier Díez-Domingo[3,5]

Affiliation

[1]NIHR Health Protection Research Unit (HPRU) in Healthcare Associated Infections (HCAI) and Antimicrobial Resistance (AMR) at Imperial College London, London, United Kingdom.

[2]Department of Obstetrics and Gynaecology, Hospital Universitario de la Ribera, Valencia, Spain

[3]Faculty of Nursing, Universidad Católica de Valencia 'San Vicente Mártir', Valencia, Spain.

[4]Xàtiva-Ontinyent Health Department, Xàtiva, Spain.

[5]Foundation for the Promotion of Health and Biomedical Research in the Valencian Region (FISABIO).

[6]Department of Nursing, University of Alicante. San Vicente del Raspeig, Alicante. Spain.

Corresponding author

Enrique Castro-Sánchez, Imperial College London, Du Cane Road, London W12 0NN, UK, [e.castro-sanchez@imperial.ac.uk], +44 203 313 2732.

Word count: 2871

For peer review only

For peer review only - http://bmjopen.bmj.com/site/about/guidelines.xhtml

ABSTRACT

Objectives

Immunizations against influenza and *Bordetella pertussis* infection are recommended to pregnant women in Valencia (Spain), yet vaccination rates remain low. Health literacy (HL) appears as a crucial factor in vaccination decision-making. We explored the relation between HL of pregnant women and decisions to receive influenza and pertussis immunizations.

Setting

University hospital in Valencia (Spain).

Participants

119 women who gave birth at a hospital in Valencia (Spain) between November 2015 and May 2016. Women in the immediate postpartum period (more than 27 weeks of gestation), between November 2015 and May 2016 were included in the study. Women with impairments, language barriers or illiteracy which prevented completion of the questionnaires, or those who were under 18 years were excluded from enrolment.

Primary and secondary outcome measures: health literacy level; influenza and pertussis immunisation rate; reasons for rejection of vaccination.

Results

119 participants were included (mean age 32.3 ± 5.5 years, 52% primiparous, 95% full term deliveries). A higher education level was associated with SAHLSA_50 (adjusted R-squared= 0.22, p=0.014) and NVS (adjusted R-squared=0.258, p=0.001) scores. Depending on the scale, 56%-85% of participants had adequate HL. 52% (62/119) and 94% (112/119) of women received influenza and pertussis immunization, respectively. Women rejecting influenza vaccine had a higher HL level (measured by SALHSA_50 tool) than those accepting it (Kruskal-Wallis test p=0.022). 24% of women who declined influenza

For peer review only - http://bmjopen.bmj.com/site/about/guidelines.xhtml

vaccination felt the vaccine was unnecessary, and 23% claimed to have insufficient information.

Conclusions

Influenza vaccination rate was suboptimal in our study. Women with high HL were more likely to decline immunization. Information from professionals needs to match patients' HL levels to reduce negative perceptions of vaccination.

Keywords: influenza, pertussis, immunisation, health literacy, maternal medicine

For peer review only

STRENGTHS AND LIMITATIONS OF THE STUDY

- This study investigated the relation between health literacy and immunisations routinely offered to pregnant women in Spain.
- Health literacy screening tools were administered to pregnant women to identify health literacy levels. Influenza and pertussis immunisation status was obtained from official vaccination records. Vaccination rates were compared between women with adequate/inadequate health literacy.
- The results highlighted the impact of both low and high health literacy on immunisation decision-making, suggesting the need for careful vaccination support messages from healthcare professionals.
- Although screening tools used in the study have been validated in Spanish-speaking populations in the US, they have not been applied in Spain.
- Further research could consider the development and use of pregnancy-specific scales.

For peer review only

BACKGROUND

Despite its benefits, influenza vaccine coverage among pregnant women remains low.[1] Some determinants associated with vaccination rejection include insufficient information by professionals and underestimation of infection risks during pregnancy.[2–4]

However, pregnancy is a risk factor for severe influenza, a main reason for hospital admission during gestation.[5] The administration of influenza vaccine to pregnant women would protect immunized mothers and infants. As the safety of the vaccine is well established, its administration is recommended during any trimester of gestation. Globally, influenza vaccination coverage is uneven, ranging from 15-43% in Europe,[6] to 50% in United States (US).[7] In Spain there are no published data on national influenza vaccination coverage among pregnant women; however, our review in 2014-5 reported vaccination rates of 40.5% in pregnant women in our health department.[8]

Vaccination against *Bordetella pertussis* is equally recommended to all pregnant women in Valencia (Spain) since January 2015 due to outbreaks of whooping cough.[9] Women are offered immunisation on the third trimester, ideally between weeks 27-36 of gestation.[6] As with influenza, maternal immunization also benefits newborns.[10] According to WHO, 195,000 children under 5-years died in 2008 of whooping cough. More than 80% of deaths occurred in children younger than 6 months of age. The number of whooping cough cases has increased since 2011 worldwide, including the European Union, and among children and young adults. In Spain, the case incidence has shifted from 739 cases in 2008 to 3,088 cases in 2011, a global rate of 6.73/100,000 hab/year for that year. Additionally, 8 deaths in 2001 were attributed to whooping cough[11]. Of concern, there are currently no published data regarding whooping cough vaccination coverage among pregnant women in

For peer review only - http://bmjopen.bmj.com/site/about/guidelines.xhtml

Spain. However, reports on the incidence of whooping cough in 2015 are available indicating 17.99 cases per 100,000 people, with provisional data for 2016 suggesting a marked decline in reported cases.[12]

Among the factors determining vaccination acceptance, health literacy (HL) refers to the knowledge and skills required when making health decisions.[13] Essential HL skills include reading, writing, numeracy, and searching for information.[14,15] Inadequate HL has been associated with poor health outcomes including inadequate self-caring and preventive behaviours such as vaccination.[16] Standardized tools for assessing HL are available, yet mostly in English[17] and focused on US society. European researchers have developed questionnaires,[18] and some tools (Short Assessment of Health Literacy for Spanish Adults (SAHLSA_50),[19,20] Newest Vital Sign (NVS) [21–24] and Single Item Literacy Screener (SILS)[25] have been validated in Spanish language but not for Spanish citizens.

Although vaccination is especially relevant for pregnant women and wider public health,[26] no studies have been conducted in Spain exploring the relationship between HL and vaccine acceptance. We hypothesise that pregnant women with limited health literacy may be less likely to accept influenza and pertussis vaccinations in Valencia (Spain).

METHODS

Study population and sampling criteria

We conducted a cross-sectional study in women who had given birth at *La Ribera* university hospital (*Hospital Universitario de La Ribera,* HULR) in Valencia (Spain). The HULR serves a population of 250,000 citizens and is the only hospital providing maternity services to pregnant women in the area. The influenza and pertussis vaccine policy in the HULR mirrors the national policy, where vaccines are offered systematically, by community midwives and family doctors, to all women free of charge.

For peer review only - http://bmjopen.bmj.com/site/about/guidelines.xhtml

Immunization campaign in Spain starts in October and concludes in March. In order to avoid seasonality, we included all women during the study period. Women in their immediate postpartum period (more than 27 weeks of gestation), between November 2015 and May 2016 were included in the study. We excluded women with impairments, language barriers or illiteracy. Illiterate women were excluded from the study due to their inability to complete the health literacy screening tools, which were self-administered. Any help from the researchers would likely influence the results.[27] Women younger than 18 years were also excluded from taking part. Prior to data collection, written consent was obtained from each participant. The study was conducted according to the basic principles of the Declaration of Helsinki for all medical research and it was approved by the research ethics and research committee of HULR on 10/07/15.

For recruitment, we systematically approached all women admitted to the maternity ward, every 4 days. To calculate the sample size, we used the SALHSA_50 tool as a reference with a cut-off score of 0-37 for inadequate literacy. Accepting an alpha risk of 0.05 and a beta risk of 0.2 in a bilateral contrast, with a common standard deviation of 7.0[28] and a loss to follow-up rate of 10%, we estimated that 102 participants would be required.

Measurements

During the immediate postpartum (24-48 hours after delivery), we collected sociodemographic, obstetric variables and vaccination status through review medical records, as well as health literacy from each woman through interview with the researcher in charge.

Participants' HL was determined using three screening tools:

1) SALHSA_50: Evaluates word recognition and reading comprehension through a 50-item tool. Quantitative scores classify individuals with "adequate" (score: 37-50 points) or "inadequate" HL (score: 0-37 points). The tool has been validated for Hispanics in the US.

For peer review only - http://bmjopen.bmj.com/site/about/guidelines.xhtml

2) NVS: Evaluates reading and numeracy through six questions about the label of an ice-cream. The sum score (0–6 points) categorize individuals with high likelihood limited literacy (score: 0-1 points), possibility of limited literacy (score: 2-3 points), and adequate literacy (score: 4-6 points). It has been validated for the Hispanic population in the US.[23] It has high sensitivity, but it can misclassify people with adequate health literacy.[29]

3) SILS: It asks patients how often they need help when reading health instructions. The response is recorded on a 5-point Likert-type scale (1-Never, 2-Rarely, 3-Sometimes, 4-Often and 5-Always) and categorized as adequate or inadequate. Scores greater than 2 indicate some difficulty with reading materials.[25]

Regarding vaccination, we analysed: 1) influenza or pertussis vaccination status during pregnancy; 2) if vaccinated, health centre where vaccinated, 3) which healthcare provider recommended it and; 4) if vaccination rejection, reasons for declining. Vaccination status was corroborated using the regional Vaccination Registry which records all vaccines received by patients. [30]

Other variables collected through review of medical records included: age, country of origin, civil status, occupation, education, gestational age, parity, type of delivery, risk factors during pregnancy (without risk or low risk, pre-gestational or gestational diabetes, thyroid pathology, preeclampsia, twin pregnancy, and assisted reproduction treatment).

Statistical analysis

In the univariate analysis, quantitative variables were described with means and standard deviation or median and interquartile range, depending on the normality of their distribution. The Kolmogorov-Smirnov goodness-of-fit test was used to determine the normality of distributions. In the bivariate analysis, the Chi-square test was used between the qualitative variables and the vaccination status. To compare the medical risk factors during pregnancy

For peer review only - http://bmjopen.bmj.com/site/about/guidelines.xhtml

related to vaccination, odds ratio (OR) with a 95% Confidence Interval (95% CI) were calculated.

The non-parametric Mann-Whitney U test was used when the normality hypothesis was rejected when comparing independent samples with the categorised values of NVS and SAHLSA_50 and vaccination acceptance. To identify the variables explaining the level of HL according to each screening tool a series of multivariate analyses were conducted. The multivariate lineal regression analysis (Wald statistic) was used regarding the explanatory covariates for the quantitative tools NVS and SALHSA_50, and a multinomial model was constructed for the qualitative scale SILS. The level of statistical significance was set at 0.05. The statistical package SPSS for Windows, V.22.0 (IBM Corp. Armonk, NY: IBM Corp) was used for data analysis.

RESULTS

Out of a total of 168 women who initially consented to be included in the study 49 were excluded (29%) for the following reasons: 10 (20%) were breastfeeding, 16 (33%) had language barriers, 16 (33%) were busy, 4 (8%) were absent from their room and 3 (6%) were unwell. Therefore, the study sample comprised 119 participants (71%).

Table 1 presents sociodemographic characteristics of participants. The mean age was 32.3 ± 5.5 years, with 29.5 ± 5.4 as mean age for the first pregnancy. 52% (62) were primiparous. The mean gestational age at delivery was 39.1 ± 1.5, with 95% (113) full term deliveries (37-42 weeks).

For peer review only - http://bmjopen.bmj.com/site/about/guidelines.xhtml

Table 1. Socio-demographic, clinical and obstetric characteristics of the sample by vaccine influenza status (n=119)

		Total row	Unvaccinated n=57		Vaccinated n=62		[a]p-value
		N	N	%	N	%	
Civil Status	With partner	48	20	35	28	45	0.458
	Married/civil partner	67	35	61	32	52	
	Separated/Divorced	4	2	4	2	3	
Level of Education	Primary school	40	20	36	20	32	0.296
	Secondary school	42	19	33	23	37	
	University	37	18	44	19	31	
Employment Status	I	13	9	16	4	6	0.083
	II	66	35	61	31	50	
	III	2	0	0	2	3	
	IV	1	0	0	1	2	
	V	37	13	23	24	39	
Country of Origin	Spain	104	51	89	53	85	0.261
	Another EU country	8	5	9	3	5	
	Non-EU country	1	0	0	1	2	
	Central-South America	6	1	2	5	8	
Pertussis vaccine	Unvaccinated	7	7	12	0	0	0.269
	Vaccinated	112	50	88	62	100	
Medical Risk Factors During Pregnancy	None/Low risk	92	45	79	47	76	0.570
	Pre/Gest Diabetes	7	2	3	5	8	
	Thyroid pathology	7	5	9	2	3	
	Preeclampsia	1	0	0	1	2	
	Twin pregnancy	3	1	2	2	3	
	ART	9	4	7	5	8	
NVS categories	Inadequate (0-1 points)	13	6	10	7	11	0.219
	Limited (2-3 points)	38	14	25	24	39	
	Adequate (4-6 points)	68	37	65	31	50	
SAHLSA categories	Inadequate (0-37 points)	17	6	10	11	18	0.261
	Adequate (38-50 points)	102	51	89	51	82	
SILS categories	Never	29	13	23	16	26	0.947
	Rarely	34	17	30	17	27	
	Sometimes	33	17	30	16	26	
	Often	8	4	7	4	6	
	Always	15	6	10	9	14	

For peer review only - http://bmjopen.bmj.com/site/about/guidelines.xhtml

[a]Chi-square; I: self-employed, higher professional or managerial employment; II: employee; III: student; IV: stay-at-home mother; V: unemployed; EU= European Union; ART= Assisted Reproduction Treatment.

The information and recommendation about vaccination came mainly from their midwives (94%), in 4% from the family doctor and 2% of women did not provide any information. As we wanted to be as sure as possible of the vaccination status of each participant, we validated the vaccination status reported by the participants with the immunization status recorded in the official electronic immunization registry. We corroborated that all women without immunisation recorded on the electronic record had not been vaccinated.

Regarding HL screening tools, the correlation between SAHLSA_50 and SILS was moderate, inversely proportional and significant (r= -0.251, p=0.007). The correlation between NVS and SAHLSA_50 was moderate and significant (r= 0.349, p<0.001). The correlation between NVS and SILS was moderate, inversely proportional and also significant (r= -0.307, p=0.001).

We also analysed the influence of participants' education on HL level and the scales of assessment. Higher education was directly related to higher SAHLSA_50 (r= 0.244, p<0.001) and NVS (r= 0.366, p=0.002) scores. This relationship however was not present in the SILS scale.

Vaccination status

17% (20/62) of women had been vaccinated against influenza prior to pregnancy. Gestational influenza vaccination coverage was 52% (62/119). The vaccine was administered to 5% (4/62) of women by week 20, and to 16% (10/62) in the last weeks of gestation (more than 36 weeks). Concerning pertussis vaccine, 94% (112/119) of women had it during pregnancy, with 86% (96/112) vaccinated between weeks 27-32 of gestation. All women vaccinated against influenza were simultaneously vaccinated against whooping cough. There were no

For peer review only - http://bmjopen.bmj.com/site/about/guidelines.xhtml

significant differences in sociodemographic or obstetric characteristics between pregnant vaccination status for influenza or pertussis (p=0.15 and p=0.35, respectively) [data not shown].

The reasons for rejection of women who were not vaccinated against influenza during pregnancy (57) are shown in **Figure 1**. 25% (14/57) felt that the vaccine was unnecessary, 23% (13/57) claimed to have received insufficient information from health professionals and 14% (8/57) claimed that they had never been infected. The reasons reported by women declining vaccination against pertussis were lack of information from health professionals (4 [57%]) and lack of any prenatal care (3 [43%]).

Figure 1 about here

Health literacy

In the NVS scale we obtained an average score of 3.7 ± 1.6 with values between 0 and 6. These scores were categorized as inadequate [13% (16/119)], limited [30% (36/119)] and adequate HL [56% (67)]. SAHLSA_50 scores were 44.1 ± 4.4 out of 50. An 86% (102/119) of women had adequate HL levels (SAHLSA-50 score>37). According to the SILS, 24% (29/119) women replied ‘never’ needing help when reading information, 29% (35/119) ‘rarely’, 27% (32/119) ‘sometimes’ and only 6% (7/119) replied ‘often’ and 13% (16/119) replied ‘always’.

To identify variables explaining HL levels according to each screening tool multivariate analyses were conducted. Multivariate lineal regression was used regarding the explanatory covariates for quantitative tools NVS and SALHSA_50. For these, the level of education was found to be statistically significant (NVS [adjusted R-squared=0.258;

For peer review only - http://bmjopen.bmj.com/site/about/guidelines.xhtml

p=0.001] and SALHSA_50 [adjusted R-squared=0.220; p=0.014]). A multinomial model was constructed for the qualitative scale SILS, observing no statistically significant differences.

There were no differences in NVS and SILS scores between women who declined and those who accepted influenza vaccination (Mann-Whitney U test, p=0.320 and p=0.942 respectively). However, for SAHLSA_50 (Median=44.5; IQR=5.0 *vs* 45.0; IQR=5.5) the differences were statistically significant (Mann-Whitney U test, p=0.019) (See **Figure 2).**

Figure 2 about here

Later, scores from the quantitative health literacy screening tools (NVS, SALHSA_50) were distributed in quartiles (**Figure 3**). For the NVS scale we found no statistically significant different between women who had accepted or declined vaccination (p=0.532). However, such difference was seen when using the SALHSA_50 tool (Kruskal-Wallis test, p=0.022). The median number of women vaccinated in the bottom quartile was 8 (95% CI: 7.0-9.0) versus 24 (95% CI: .23.0-25.0) in the top quartile.

We were interested in examining the characteristics of the women who were excluded from the study (49). We conducted an analysis of missing values for the three health literacy screening tools using the multiple imputation chained equations method.[31] Again, for the NVS scale we found no statistically significant difference between women who had accepted or declined vaccination (p=0.372) and, instead, such difference was seen when using the SALHSA_50 tool (Kruskal-Wallis test, p=0.003). The median number of women vaccinated in the bottom quartile was 11 (95% CI: 9.0-12.0) vs 28 (95% CI: 27.0-29.0) in the top quartile.

Figure 3 about here

For peer review only - http://bmjopen.bmj.com/site/about/guidelines.xhtml

Regarding the NVS, scores between pertussis-vaccinated and unvaccinated women were similar (Median= 4.0; IQR=0.0 *vs* Median=4.0; IQR=2.75), like the SAHLSA_50 scale (Median= 45.0; IQR=0.0 *vs* Median= 45.0; IQR=5.0). We also did not find any difference with the results from the SILS tool.

DISCUSSION

Vaccination is an essential public health intervention. We focused on pregnant women, an especially vulnerable population, and studied the acceptance of two vaccines underutilised in our community.[8] Few studies evaluating HL and vaccination have been conducted thus far and, up to now, none had focused on pregnant women.

In our study, influenza vaccination did not reach recommended levels although coverage slightly exceeded Australian[32] but not US rates.[33] In Valencia coverage has progressively improved from 2011 (8.5%) to 2015 (34.4%).[8] Regarding pertussis, the 97% vaccination rate improves on Belgian (39%)[34] or UK (70%)[6] rates. We believe that fear to pertussis, influenced by mass media [35] could explain such high vaccination prevalence. The disparity between pertussis and influenza immunization rates has not been previously addressed.[6] The disinterest from health professionals together with maternal perceptions that influenza vaccine was unnecessary were the most frequently cited causes of vaccine rejection, in agreement with prior studies.[5–8,36] This position obviously ignores the benefits of acquired immunity for the newborn, which could reduce perinatal infections.[37]

We found that NVS classified 58% of participants with adequate HL. However, this figure increased up to 89% if SAHLSA_50 was used. Currently, there are no publications comparing both scales simultaneously in the same population. Such discrepancy between screening tools could be of much relevance as, of the tools pragmatically chosen for our

For peer review only - http://bmjopen.bmj.com/site/about/guidelines.xhtml

research, only SALHSA_50 was predictive of vaccination in pregnant women. However, women with high SALHSA_50 scores were more likely to decline influenza vaccination, perhaps due to pre-conceived ideas; it might also be that women with high HL have more abilities to look for information on the internet and are deceived by the information they found and as such decline vaccination,[38] or failures by professionals to adequately inform them.[37,39,40] These results diverge from current evidence[4] in this group of women possibly highly involved in their health care, as already explored.[41]

As perhaps expected, HL screening results were directly related to the education of participants and thus, a higher level of education was associated with higher HL. Interestingly, other authors have reported that a higher level of education is associated with higher rates of vaccine rejection and hesitation.[36,42] In fact, it would appear that the emerging relationship between HL and vaccination described by those authors may be represented as an 'inverted U' shape' (i.e., high and low HL levels equally associated with low vaccination).

The analysis of missing values would help resolve some of the challenges originated from the incomplete responses. If cases with missing data were to be systematically different to cases with complete information, then results could be equivocal.[31] In our case, however, the analysis of missing values did not produce different results to the original analysis conducted without imputed values.

Our study presents limitations. Although there are approximately 51 HL tools available,[17] experiences in Spain with these instruments have been few and limited to the Health Literacy Survey - European Union[43] or the eHealth Literacy (eHL)[44] tools. In addition, none of these tools have been validated in Spain, yet they have been so in Spanish-speaking US populations. Moreover, as there are no scales specifically focused on pregnant women, our questionnaire selection was eminently pragmatic and based on ease of use (SILS), robustness (SAHLSA_50) and reliability (NVS). Additionally, the routine use of HL

For peer review only - http://bmjopen.bmj.com/site/about/guidelines.xhtml

screening tools remains nevertheless controversial, as routine screening has shown no benefits yet could have undesirable effects.[45]

Decisions related to vaccination may be influenced by the information provided, the communication approaches and attitudes of by health professionals.[36,37] Since there is currently no standardized approach to determine the abilities that pregnant women have to make effective use of the information provided, we hypothesise that information offered to each woman will be more or less similar and, therefore, women with low HL may be more likely to make suboptimal decisions because of such deficit. Logically, this does not consider efforts that professionals may make to compensate for any difficulties in understanding. Although exploring such efforts was outside the remit of our work, it would be interesting to investigate this aspect in future studies, together with any supporting materials used by professionals.

CONCLUSION

Vaccination is an essential public health measure and pregnant women can particularly benefit from this intervention. Identifying determinants of vaccination such as health literacy would facilitate an adequate use of resources to encourage shared decision-making, ultimately resulting in optimal vaccination rates. Our findings suggesting a relation between high health literacy and rejection of vaccination encourage further research to identify and describe the factors involved in such relation and implement mitigating initiatives.

For peer review only - http://bmjopen.bmj.com/site/about/guidelines.xhtml

WHAT IS ALREADY KNOWN ON THE SUBJECT

- Vaccination coverage against influenza and whooping cough among pregnant women worldwide remains suboptimal.
- Health literacy influences healthcare decisions including vaccination.
- There is a lack of studies conducted in Spain exploring the relationship between HL and vaccine acceptance among pregnant women.

WHAT THIS STUDY ADDS

- Only half of pregnant women in Spain accepted influenza vaccination.
- Women with high health literacy were more likely to reject vaccination.
- Provision of information about immunisation together with vaccination narratives tailored to health literacy levels of women remain unresolved matters.

For peer review only

For peer review only - http://bmjopen.bmj.com/site/about/guidelines.xhtml

FUNDING

This work was supported by the Universidad Católica de Valencia "San Vicente Mártir" (Spain) through a competitive grant call [PRUCV/2015/639]. ECS is affiliated with the National Institute for Health Research (NIHR) Health Protection Research Unit (HPRU) in Healthcare Associated Infection and Antimicrobial Resistance at Imperial College London in partnership with Public Health England (PHE), and the NIHR Imperial Patient Safety Translational Research Centre. ECS has received an Early Career Research Fellowship from the Antimicrobial Research Collaborative at Imperial College London and acknowledges the support of the Florence Nightingale Foundation. The views expressed are those of the authors and not necessarily those of the NHS, the NIHR, the Department of Health, or Public Health England. The funders had no role in study design; collection, analysis, and interpretation of data; writing the report; and the decision to submit the report for publication.

COMPETING INTERESTS

None declared.

AUTHOR CONTRIBUTION

ECS RVC conceptualized and designed the study, RVC FSV collected data, ECS RVC FSV carried out the data analyses, ECS RVC ENI JDD FSV drafted the initial manuscript, ECS RVC FSV ENI JDD reviewed and revised the manuscript. All authors read and approved the final manuscript.

For peer review only - http://bmjopen.bmj.com/site/about/guidelines.xhtml

DATA SHARING

No additional data are available.

ETHICAL APPROVAL

The study was conducted in compliance with the Declaration of Helsinki and it was approved by the research ethics and research committee of HULR on 10/07/15.

ACKNOWLEDGEMENTS

We are grateful to the Universidad Católica de Valencia and to the HULR for their support and participation in this project, and to all the women who have also taken part. We also thank Professor Daniel Lee for authorising the use of the SALHSA_50 tool in our research.

For peer review only

REFERENCES

1. Fiore AE, Uyeki TM, Broder K, et al. Prevention and control of influenza with vaccines: recommendations of the Advisory Committee on Immunization Practices (ACIP), 2010. *MMWR Recomm Rep*. 2010;59(RR-8):1-62. doi:10.1111/j.1600-6143.2011.03793.x.

2. Regan AK, Mak DB, Hauck YL, Gibbs R, Tracey L, Effler P V. Trends in seasonal influenza vaccine uptake during pregnancy in Western Australia: Implications for midwives. *Women and Birth*. 2016;29(5):423-429. doi:10.1016/j.wombi.2016.01.009.

3. Tamma PD, Ault KA, del Rio C, Steinhoff MC, Halsey NA, Omer SB. Safety of influenza vaccination during pregnancy. *Am J Obstet Gynecol*. 2009;201(6):547-552. http://www.ajog.org/article/S0002-9378(09)01108-9/pdf.

4. Yudin MH, Salaripour M, Sgro MD. Pregnant Women's Knowledge of Influenza and the Use and Safety of the Influenza Vaccine During Pregnancy. *J Obstet Gynaecol Canada*. 2009;31(2):120-125. doi:10.1016/S1701-2163(16)34095-6.

5. Panda B, Stiller R, Panda A. Influenza vaccination during pregnancy and factors for lacking compliance with current CDC guidelines. *J Matern Fetal Neonatal Med*. 2011;24(3):402-406. doi:10.3109/14767058.2010.497882.

6. Laenen J, Roelants M, Devlieger R, Vandermeulen C. Influenza and pertussis vaccination coverage in pregnant women. *Vaccine*. 2015;33(18):2125-2131. doi:10.1016/j.vaccine.2015.03.020.

7. Ding H, Black CL, Ball S, et al. Influenza Vaccination Coverage Among Pregnant Women--United States, 2014-15 Influenza Season. *MMWR Morb Mortal Wkly Rep*.

For peer review only - http://bmjopen.bmj.com/site/about/guidelines.xhtml

2015;64(36):1000-1005. doi:10.15585/mmwr.mm6436a2.

8. Vila-Candel R, Navarro-Illana P, Navarro-Illana E, et al. Determinants of seasonal influenza vaccination in pregnant women in Valencia, Spain. *BMC Public Health*. 2016;16:1173. doi:10.1186/s12889-016-3823-1.

9. Torres J, Godoy P, Artigues A, et al. [Outbreak of whooping cough with a high attack rate in well-vaccinated children and adolescents]. *Enfermedades Infecc y Microbiol clínica*. 2011;29(8):564-567. doi:10.1016/j.eimc.2011.04.005.

10. Amirthalingam G, Andrews N, Campbell H, et al. Effectiveness of maternal pertussis vaccination in England: An observational study. *Lancet*. 2014;384(9953):1521-1528. doi:10.1016/S0140-6736(14)60686-3.

11. WHO. Pertussis vaccines: WHO position paper, August 2015-Recommendations. *Vaccine*. 2016;34(12):1423-1425. doi:10.1016/j.vaccine.2015.10.136.

12. Situación de la Tos ferina en España, 2005-2016. Centro Nacional de Epidemiología. Minesterio de Economía, Industria y Competitividad. http://www.isciii.es/ISCIII/es/contenidos/fd-servicios-cientifico-tecnicos/fd-vigilancias-alertas/fd-enfermedades/pdf_2016/INFORME_Tos_ferina_Espana_2005-2016.pdf. Published 2016. Accessed October 20, 2017.

13. Sørensen K, Van den Broucke S, Fullam J, et al. Health literacy and public health: A systematic review and integration of definitions and models. *BMC Public Health*. 2012;12(1):80. doi:10.1186/1471-2458-12-80.

14. Baker DW. The meaning and the measure of health literacy. *J Gen Intern Med*. 2006;21(8):878-883. doi:10.1111/j.1525-1497.2006.00540.x.

15. Nutbeam D. Health literacy as a public goal: a challenge for contemporary health education and communication strategies into the 21st century. *Heal Promot Int*. 2000;15(3):259-267. doi:10.1093/heapro/15.3.259.

For peer review only - http://bmjopen.bmj.com/site/about/guidelines.xhtml

16. Heijmans M, Uiters E, Rose T, et al. *Study on Sound Evidence for a Better Understanding of Health Literacy in the European Union.*; 2015. doi:10.2818/150402.

17. Haun JN, Valerio MA, McCormack LA, Sørensen K, Paasche-Orlow MK. Health literacy measurement: An inventory and descriptive summary of 51 instruments. *J Health Commun*. 2014;19:302-333. doi:10.1080/10810730.2014.936571.

18. Sørensen K, Pelikan JM, Röthlin F, et al. Health literacy in Europe: comparative results of the European health literacy survey (HLS-EU). *Eur J Public Health*. 2015;25(6):1053-1058. doi:10.1093/eurpub/ckv043.

19. Lee S-YD, Stucky BD, Lee JY, Rozier RG, Bender DE. Short Assessment of Health Literacy-Spanish and English: a comparable test of health literacy for Spanish and English speakers. *Health Serv Res*. 2010;45(4):1105-1120. doi:10.1111/j.1475-6773.2010.01119.x.

20. Vilca Yengle LM, Campins Martí M, Cabero Roura L, et al. [Influenza vaccination in pregnant women. Coverage, practices and knowledge among obstetricians]. *Med Clin (Barc)*. 2010;134(4):146-151.

21. Castro-Sánchez E, Chang PWS, Vila-Candel R, Escobedo AA, Holmes AH. Health literacy and infectious diseases: why does it matter? *Int J Infect Dis*. 2016;43:103-110. doi:10.1016/j.ijid.2015.12.019.

22. Morrison AK, Schapira MM, Hoffmann RG, Brousseau DC. Measuring health literacy in caregivers of children: a comparison of the newest vital sign and S-TOFHLA. *Clin Pediatr (Phila)*. 2014;53(13):1264-1270. doi:10.1177/0009922814541674.

23. Warsh J, Chari R, Badaczewski A, Hossain J, Sharif I. Can the Newest Vital Sign be used to assess health literacy in children and adolescents? *Clin Pediatr (Phila)*. 2014;53(2):141-144. doi:10.1177/0009922813504025.

24. Weiss BD, Mays MZ, Martz W, et al. Quick assessment of literacy in primary care:

For peer review only

The newest vital sign. *Ann Fam Med*. 2005;3(6):514-522. doi:10.1370/afm.405.

25. Morris NS, MacLean CD, Chew LD, Littenberg B. The Single Item Literacy Screener: evaluation of a brief instrument to identify limited reading ability. *BMC Fam Pract*. 2006;7:21. doi:10.1186/1471-2296-7-21.

26. Mak TK, Mangtani P, Leese J, Watson JM, Pfeifer D. Influenza vaccination in pregnancy: current evidence and selected national policies. *Lancet Infect Dis*. 2008;8(1):44-52.

27. Davis RE, Couper MP, Janz NK, Caldwell CH, Resnicow K. Interviewer effects in public health surveys. *Health Educ Res*. 2010;25(1):14-26. http://dx.doi.org/10.1093/her/cyp046.

28. Padilla-Santoyo P, Vílchez-Román C. Psychometric properties of the SAHLSA-50, a standardized test to evaluate the health literacy. *Rev Per Obs Enf*. 2008;4(2):90-95.

29. Delanoë A, Lépine J, Leiva Portocarrero ME, et al. Health literacy in pregnant women facing prenatal screening may explain their intention to use a patient decision aid: a short report. *BMC Res Notes*. 2016;9(1):339. doi:10.1186/s13104-016-2141-0.

30. Vila-Candel R, Navarro-Illana P, Navarro-Illana E, et al. Determinants of seasonal influenza vaccination in pregnant women in Valencia, Spain. *BMC Public Health*. 2016;16(1):1173. doi:10.1186/s12889-016-3823-1.

31. Hayati Rezvan P, Lee KJ, Simpson JA. The rise of multiple imputation: a review of the reporting and implementation of the method in medical research. *BMC Med Res Methodol*. 2015;15(1):30. doi:10.1186/s12874-015-0022-1.

32. Maher L, Hope K, Torvaldsen S, et al. Influenza vaccination during pregnancy: Coverage rates and influencing factors in two urban districts in Sydney. *Vaccine*. 2013;31(47):5557-5564. doi:10.1016/j.vaccine.2013.08.081.

33. Henninger M. Trends in Influenza Vaccine Coverage in Pregnant Women, 2008 to

For peer review only - http://bmjopen.bmj.com/site/about/guidelines.xhtml

2012. *Perm J*. 2013;17(2):31-36. doi:10.7812/TPP/12-115.

34. Maertens K, Caboré RN, Huygen K, Hens N, Van Damme P, Leuridan E. Pertussis vaccination during pregnancy in Belgium: Results of a prospective controlled cohort study. *Vaccine*. 2016;34(1):142-150. doi:10.1016/j.vaccine.2015.10.100.

35. Campins M, Moreno-Perez D, Gil-de Miguel A, et al. [Whooping cough in Spain. Current epidemiology, prevention and control strategies. Recommendations by the Pertussis Working Group]. *Enferm Infecc Microbiol Clin*. 2013;31(4):240-253. doi:10.1016/j.eimc.2012.12.011.

36. Biasio LR. Vaccine hesitancy and health literacy. *Hum Vaccin Immunother*. 2017;13(3):701-702. doi:10.1080/21645515.2016.1243633.

37. Lorini C, Santomauro F, Donzellini M, et al. Health literacy and vaccination: A systematic review. *Hum Vaccin Immunother*. October 2017:00-00. doi:10.1080/21645515.2017.1392423.

38. Stahl JP, Cohen R, Denis F, et al. The impact of the web and social networks on vaccination. New challenges and opportunities offered to fight against vaccine hesitancy. *Med Mal Infect*. 2016;46(3):117-122. doi:10.1016/j.medmal.2016.02.002.

39. Niccolai LM, Pettigrew MM. The Role of Cognitive Bias in Suboptimal HPV Vaccine Uptake. *Pediatrics*. 2016;138(4):e20161537.

40. Yaqub O, Castle-Clarke S, Sevdalis N, Chataway J. Attitudes to vaccination: A critical review. *Soc Sci Med*. 2014;112:1-11. doi:10.1016/j.socscimed.2014.04.018.

41. Fadda M, Depping MK, Schulz PJ. Addressing issues of vaccination literacy and psychological empowerment in the measles-mumps-rubella (MMR) vaccination decision-making: a qualitative study. *BMC Public Health*. 2015;15:836. doi:10.1186/s12889-015-2200-9.

42. Hak E, Sch??nbeck Y, De Melker H, Van Essen GA, Sanders EAM. Negative attitude

For peer review only - http://bmjopen.bmj.com/site/about/guidelines.xhtml

of highly educated parents and health care workers towards future vaccinations in the Dutch childhood vaccination program. *Vaccine*. 2005;23(24):3103-3107. doi:10.1016/j.vaccine.2005.01.074.

43. Sorensen K, Van den Broucke S, Pelikan JM, et al. Measuring health literacy in populations: illuminating the design and development process of the European Health Literacy Survey Questionnair (HLS-EU-Q). *BMC Public Health*. 2013;13(1):948. doi:10.1186/1471-2458-13-948.

44. Paramio Perez G, Almagro BJ, Hernando Gomez A, Aguaded Gomez JI. [Validation of the eHealth Literacy Scale (eHEALS) in Spanish University Students]. *Rev Esp Salud Publica*. 2015;89(3):329-338. doi:10.4321/s1135-57272015000300010.

45. Dickens C. Health Literacy and Nursing. *AJN, Am J Nurs*. 2013;113(6):52-57. doi:10.1097/01.NAJ.0000431271.83277.2f.

For peer review only

For peer review only - http://bmjopen.bmj.com/site/about/guidelines.xhtml

FIGURE TITLES

Figure 1. Reasons given by participants to decline influenza vaccination

Figure 2. Relationship between acceptance of influenza vaccination and SAHLSA_50 scale (N=119)

Figure 3. Relationship between acceptance of influenza vaccination and SAHLSA_50 scale distribution by quartiles (N=119)

For peer review only

For peer review only - http://bmjopen.bmj.com/site/about/guidelines.xhtml

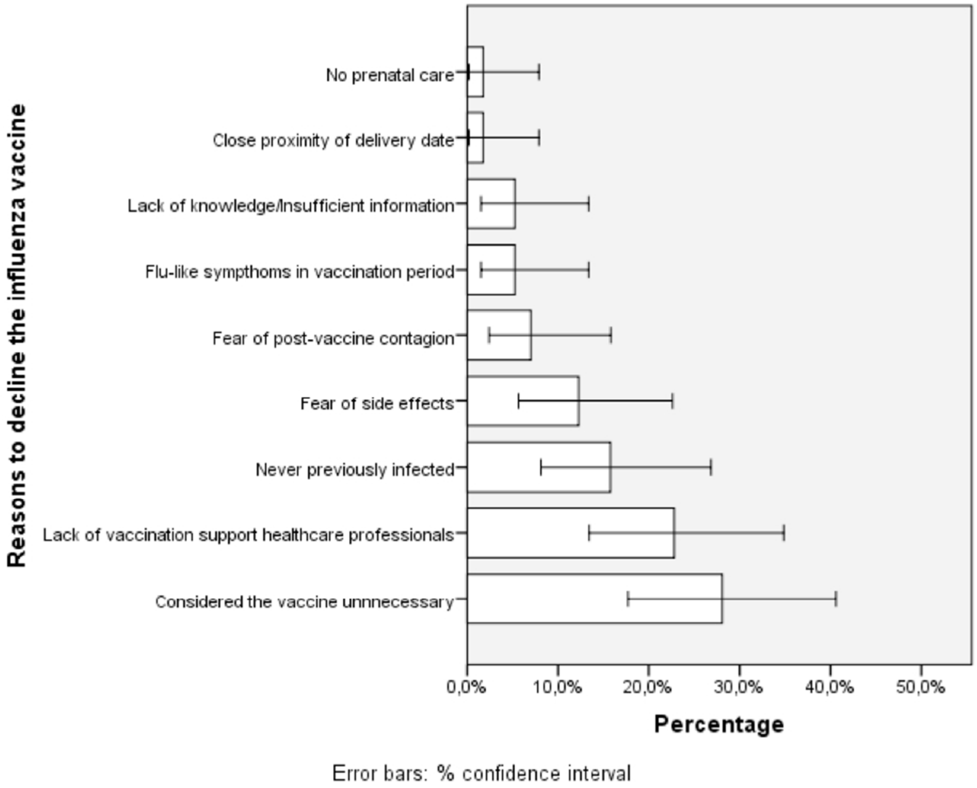

Reasons given by participants to decline influenza vaccination

159x127mm (150 x 150 DPI)

For peer review only

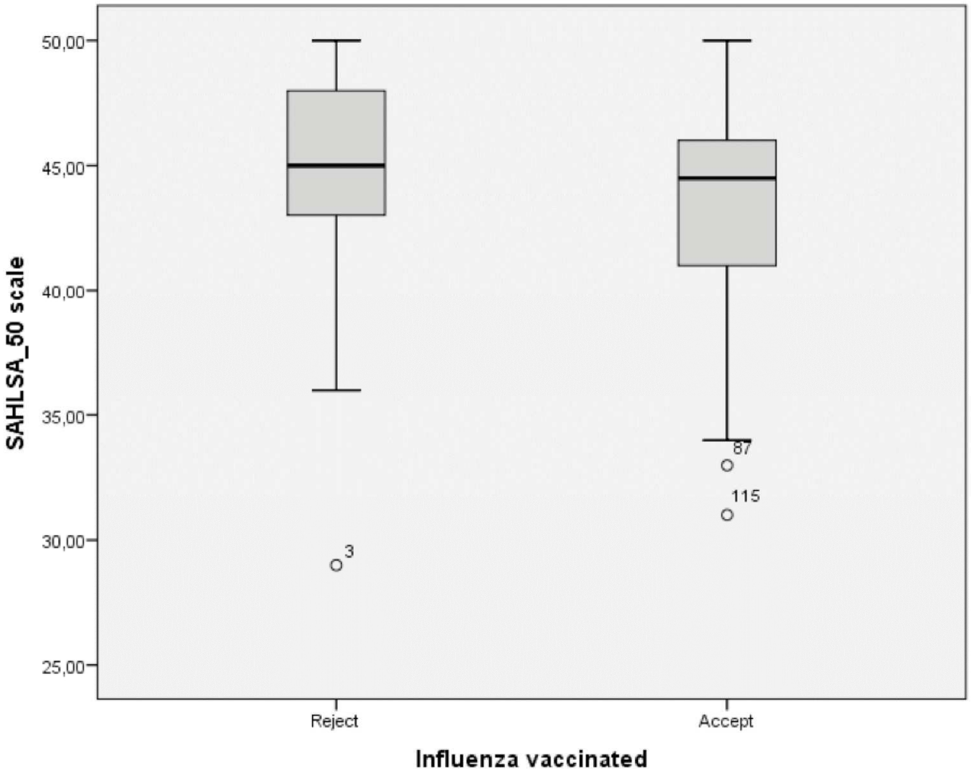

Relationship between acceptance of influenza vaccination and SAHLSA_50 scale (N=119)

497x397mm (96 x 96 DPI)

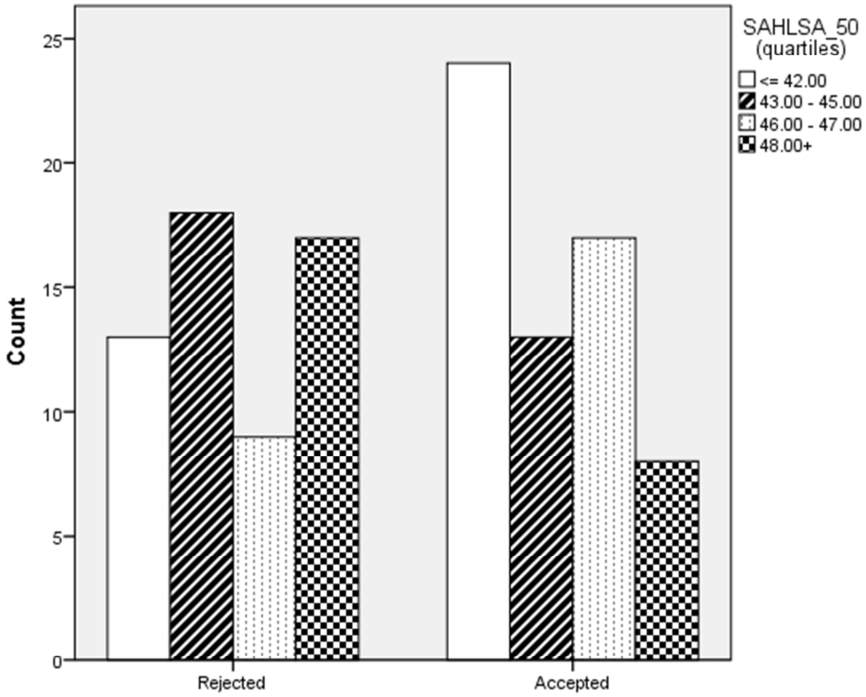

Relationship between acceptance of influenza vaccination and SAHLSA_50 scale distribution by quartiles (N=119)

165x132mm (96 x 96 DPI)

BMJ Open

Influence of health literacy on acceptance of influenza and pertussis vaccinations: a cross-sectional study among Spanish pregnant women

Journal:	*BMJ Open*
Manuscript ID	bmjopen-2018-022132.R1
Article Type:	Research
Date Submitted by the Author:	17-Apr-2018

Complete List of Authors:	Castro-Sánchez, Enrique; Imperial College London,<br>Vila-Candel, Rafael; Hospital Universitario de la Ribera, Department of Obstetrics and Gynaecology,; Universidad Católica de Valencia 'San Vicente Mártir', Faculty of Nursing<br>Soriano-Vidal, Francisco; Foundation for the Promotion of Health and Biomedical Research in the Valencian Region (FISABIO)., Xàtiva-Ontinyent Health Department; Universidad Católica de Valencia 'San Vicente Mártir', Faculty of Nursing<br>Navarro-Illana, Esther; Universidad Católica de Valencia 'San Vicente Mártir', Faculty of Nursing<br>Domingo, Javier Diez; Foundation for the Promotion of Health and Biomedical Research in the Valencian Region (FISABIO).; Universidad Católica de Valencia 'San Vicente Mártir', Faculty of Nursing

**Primary Subject Heading**:	Public health
Secondary Subject Heading:	Reproductive medicine
Keywords:	Immunisation, Influenza, Pertussis, Health literacy, Maternal medicine < OBSTETRICS

SCHOLARONE™ Manuscripts

INFLUENCE OF HEALTH LITERACY ON ACCEPTANCE OF INFLUENZA AND PERTUSSIS VACCINATIONS: A CROSS-SECTIONAL STUDY AMONG SPANISH PREGNANT WOMEN

Authors

Enrique Castro-Sánchez[1], Rafael Vila-Candel[2,3], Francisco J Soriano-Vidal[3,4,5,6], Esther Navarro-Illana[3], Javier Díez-Domingo[3,5]

Affiliation

[1]NIHR Health Protection Research Unit (HPRU) in Healthcare Associated Infections (HCAI) and Antimicrobial Resistance (AMR) at Imperial College London, London, United Kingdom.

[2]Department of Obstetrics and Gynaecology, Hospital Universitario de la Ribera, Valencia, Spain

[3]Faculty of Nursing, Universidad Católica de Valencia 'San Vicente Mártir', Valencia, Spain.

[4]Xàtiva-Ontinyent Health Department, Xàtiva, Spain.

[5]Foundation for the Promotion of Health and Biomedical Research in the Valencian Region (FISABIO).

[6]Department of Nursing, University of Alicante. San Vicente del Raspeig, Alicante. Spain.

Corresponding author

Enrique Castro-Sánchez, Imperial College London, Du Cane Road, London W12 0NN, UK, [e.castro-sanchez@imperial.ac.uk], +44 203 313 2732.

Word count: 2999

For peer review only - http://bmjopen.bmj.com/site/about/guidelines.xhtml

ABSTRACT

Objectives

Immunizations against influenza and *Bordetella pertussis* infection are recommended to pregnant women in Valencia (Spain), yet vaccination rates remain low. Health literacy (HL) appears as a crucial factor in vaccination decision-making. We explored the relation between HL of pregnant women and decisions to receive influenza and pertussis immunizations.

Setting

University hospital in Valencia (Spain).

Participants

119 women who gave birth at a hospital in Valencia (Spain) between November 2015 and May 2016. Women in the immediate postpartum period (more than 27 weeks of gestation), between November 2015 and May 2016 were included in the study. Women with impairments, language barriers or illiteracy which prevented completion of the questionnaires, or those who were under 18 years were excluded from enrolment.

Primary and secondary outcome measures: health literacy level; influenza and pertussis immunisation rate; reasons for rejection of vaccination.

Results

119 participants were included (mean age 32.3 ± 5.5 years, 52% primiparous, 95% full term deliveries). A higher education level was associated with SAHLSA_50 (adjusted R-squared= 0.22, p=0.014) and NVS (adjusted R-squared=0.258, p=0.001) scores. Depending on the scale, 56%-85% of participants had adequate HL. 52% (62/119) and 94% (112/119) of women received influenza and pertussis immunization, respectively. Women rejecting influenza vaccine had a higher HL level (measured by SALHSA_50 tool) than those accepting it (Kruskal-Wallis test p=0.022). 24% of women who declined influenza

For peer review only - http://bmjopen.bmj.com/site/about/guidelines.xhtml

vaccination felt the vaccine was unnecessary, and 23% claimed to have insufficient information.

Conclusions

Influenza vaccination rate was suboptimal in our study. Women with high HL were more likely to decline immunization. Information from professionals needs to match patients' HL levels to reduce negative perceptions of vaccination.

Keywords: influenza, pertussis, immunisation, health literacy, maternal medicine

For peer review only

STRENGTHS AND LIMITATIONS OF THE STUDY

- This study investigated the relation between health literacy and immunisations routinely offered to pregnant women in Spain.
- Validated health literacy screening tools were administered to pregnant women to identify health literacy levels. Immunisation status was obtained from official vaccination records.
- Screening tools used in the study have been validated in Spanish-speaking populations in the US but not Spain.
- It is not possible to attribute the differences in vaccinations seen to health literacy solely due to the cross-sectional study design.
- Further research could focus on the development and use of pregnancy-specific scales.

For peer review only

BACKGROUND

Despite its benefits, influenza vaccine coverage among pregnant women remains low.[1] Some determinants associated with vaccination rejection include insufficient information by professionals and underestimation of infection risks during pregnancy.[2–4]

However, pregnancy is a risk factor for severe influenza, a main reason for hospital admission during gestation.[5] The administration of influenza vaccine to pregnant women would protect immunized mothers and infants. As the safety of the vaccine is well established, its administration is recommended during any trimester of gestation. Globally, influenza vaccination coverage is uneven, ranging from 15-43% in Europe,[6] to 50% in United States (US).[7] In Spain there are no published data on national influenza vaccination coverage among pregnant women; however, our review in 2014-5 reported vaccination rates of 40.5% in pregnant women in our health department.[8]

Vaccination against *Bordetella pertussis* is equally recommended to all pregnant women in Valencia (Spain) since January 2015 due to outbreaks of whooping cough.[9] Women are offered immunisation on the third trimester, ideally between weeks 27-36 of gestation.[6] As with influenza, maternal immunization also benefits newborns.[10] According to WHO, 195,000 children under 5-years died in 2008 of whooping cough. More than 80% of deaths occurred in children younger than 6 months of age. The number of whooping cough cases has increased since 2011 worldwide, including the European Union, and among children and young adults. In Spain, the case incidence has shifted from 739 cases in 2008 to 3,088 cases in 2011, a global rate of 6.73/100,000 hab/year for that year. Additionally, 8 deaths in 2001 were attributed to whooping cough[11]. Of concern, there are currently no published data regarding whooping cough vaccination coverage among pregnant women in

For peer review only - http://bmjopen.bmj.com/site/about/guidelines.xhtml

Spain. However, reports on the incidence of whooping cough in 2015 are available indicating 17.99 cases per 100,000 people, with provisional data for 2016 suggesting a marked decline in reported cases.[12]

Among the factors determining vaccination acceptance, health literacy (HL) refers to the knowledge and skills required when making health decisions.[13] Essential HL skills include reading, writing, numeracy, and searching for information.[14,15] Inadequate HL has been associated with poor health outcomes including inadequate self-caring and preventive behaviours such as vaccination.[16] Standardized tools for assessing HL are available, yet mostly in English[17] and focused on US society. European researchers have developed questionnaires,[18] and some tools (Short Assessment of Health Literacy for Spanish Adults (SAHLSA_50),[19,20] Newest Vital Sign (NVS) [21–24] and Single Item Literacy Screener (SILS)[25] have been validated in Spanish language but not for Spanish citizens.

Although vaccination is especially relevant for pregnant women and wider public health,[26] no studies have been conducted in Spain exploring the relationship between HL and vaccine acceptance. We hypothesise that pregnant women with limited health literacy may be less likely to accept influenza and pertussis vaccinations in Valencia (Spain).

METHODS

Study population and sampling criteria

We conducted a cross-sectional study in women who had given birth at La Ribera university hospital (Hospital Universitario de La Ribera, HULR) in Valencia (Spain). The HULR serves a population of 250,000 citizens and is the only hospital providing maternity services to pregnant women in the area, with an annual average of 1600 births in the year when the study was carried out. The influenza and pertussis vaccine policy in the HULR mirrors the national policy, where vaccines are offered systematically, by community midwives and family

For peer review only

doctors, to all women free of charge. In 2015 the influenza vaccination rate for the whole Valencian Community was 34.4%.

Immunization campaign in Spain starts in October and concludes in March. In order to avoid seasonality, we included all women during the study period. Women in their immediate postpartum period (more than 27 weeks of gestation), between November 2015 and May 2016 were included in the study. We excluded women with impairments, language barriers or illiteracy. Illiterate women were excluded from the study due to their inability to complete the health literacy screening tools, which were self-administered. Any help from the researchers would likely influence the results.[27] Women younger than 18 years were also excluded from taking part. Prior to data collection, written consent was obtained from each participant. The study was conducted according to the basic principles of the Declaration of Helsinki for all medical research and it was approved by the research ethics and research committee of HULR on 10/07/15.

For recruitment, we systematically approached all women admitted to the maternity ward, every 4 days. To calculate the sample size, we used the SALHSA_50 tool as a reference with a cut-off score of 0-37 for inadequate literacy. Accepting an alpha risk of 0.05 and a beta risk of 0.2 in a bilateral contrast, with a common standard deviation of 7.0[28] and a loss to follow-up rate of 10%, we estimated that 102 participants would be required.

Measurements

During the immediate postpartum (24-48 hours after delivery), we collected sociodemographic, obstetric variables and vaccination status through review medical records, as well as health literacy from each woman through interview with the researcher in charge.

Participants' HL was determined using three screening tools:

For peer review only - http://bmjopen.bmj.com/site/about/guidelines.xhtml

1) SALHSA_50: Evaluates word recognition and reading comprehension through a 50-item tool. Quantitative scores classify individuals with "adequate" (score: 38-50 points) or "inadequate" HL (score: 0-37 points). The tool has been validated for Hispanics in the US.

2) NVS: Evaluates reading and numeracy through six questions about the label of an ice-cream. The sum score (0–6 points) categorize individuals with high likelihood limited literacy (score: 0-1 points), possibility of limited literacy (score: 2-3 points), and adequate literacy (score: 4-6 points). It has been validated for the Hispanic population in the US.[23] It has high sensitivity, but it can misclassify people with adequate health literacy.[29]

3) SILS: It asks patients how often they need help when reading health instructions. The response is recorded on a 5-point Likert-type scale (1-Never, 2-Rarely, 3-Sometimes, 4-Often and 5-Always) and categorized as adequate or inadequate. Scores greater than 2 indicate some difficulty with reading materials.[25]

Regarding vaccination, we analysed: 1) influenza or pertussis vaccination status during pregnancy; 2) if vaccinated, health centre where vaccinated, 3) which healthcare provider recommended it and; 4) if vaccination rejection, reasons for declining. Vaccination status was corroborated using the regional Vaccination Registry which records all vaccines received by patients. [30]

Other variables collected through review of medical records included: age, country of origin, civil status, occupation, education, gestational age, parity, type of delivery, risk factors during pregnancy (without risk or low risk, pre-gestational or gestational diabetes, thyroid pathology, preeclampsia, twin pregnancy, and assisted reproduction treatment).

Statistical analysis

In the univariate analysis, quantitative variables were described with means and standard deviation or median and interquartile range, depending on the normality of their distribution.

For peer review only - http://bmjopen.bmj.com/site/about/guidelines.xhtml

The Kolmogorov-Smirnov goodness-of-fit test was used to determine the normality of distributions. In the bivariate analysis, the Chi-square test was used between the qualitative variables and the vaccination status. To compare the medical risk factors during pregnancy related to vaccination, odds ratio (OR) with a 95% Confidence Interval (95% CI) were calculated.

The non-parametric Mann-Whitney U test was used when the normality hypothesis was rejected when comparing independent samples with the categorised values of NVS and SAHLSA_50 and vaccination acceptance. To identify the variables explaining the level of HL according to each screening tool a series of multivariate analyses were conducted. The multivariate lineal regression analysis (Wald statistic) was used regarding the explanatory covariates for the quantitative tools NVS and SALHSA_50, and a multinomial model was constructed for the qualitative scale SILS. The level of statistical significance was set at 0.05. SPSS for Windows, V.22.0 (IBM Corp. Armonk, NY: IBM Corp) was used for data analysis.

Patient and Public Involvement

Patients were not involved in the development of the research questions, the design of the study, or the recruitment of participants. Aggregated study results will be published on the website of the hospital, in suitable language.

RESULTS

Out of a total of 168 women who initially consented to be included in the study 49 were excluded (29%) for the following reasons: 10 (20%) were breastfeeding, 16 (33%) had language barriers, 16 (33%) were busy, 4 (8%) were absent from their room and 3 (6%) were unwell. Therefore, the study sample comprised 119 participants (71%).

For peer review only - http://bmjopen.bmj.com/site/about/guidelines.xhtml

Table 1 presents sociodemographic characteristics of participants. The mean age was 32.3 ± 5.5 years, with 29.5 ± 5.4 as mean age for the first pregnancy. 52% (62) were primiparous. The mean gestational age at delivery was 39.1 ± 1.5, with 95% (113) full term deliveries (37-42 weeks).

Table 1. Socio-demographic, clinical and obstetric characteristics of the sample by vaccine influenza status (n=119)

		**Total row**	**Unvaccinated n=57**		**Vaccinated n=62**		**[a]p-value**
		N	N	%	N	%	
Civil Status	With partner	48	20	35	28	45	0.458
	Married/civil partner	67	35	61	32	52	
	Separated/Divorced	4	2	4	2	3	
Level of Education	Primary school	40	20	36	20	32	0.296
	Secondary school	42	19	33	23	37	
	University	37	18	44	19	31	
Employment Status	I	13	9	16	4	6	0.083
	II	66	35	61	31	50	
	III	2	0	0	2	3	
	IV	1	0	0	1	2	
	V	37	13	23	24	39	
Country of Origin	Spain	104	51	89	53	85	0.261
	Another EU country	8	5	9	3	5	
	Non-EU country	1	0	0	1	2	
	Central-South America	6	1	2	5	8	
Pertussis vaccine	Unvaccinated	7	7	12	0	0	0.269
	Vaccinated	112	50	88	62	100	
Medical Risk Factors During Pregnancy	None/Low risk	92	45	79	47	76	0.570
	Pre/Gest Diabetes	7	2	3	5	8	
	Thyroid pathology	7	5	9	2	3	
	Preeclampsia	1	0	0	1	2	
	Twin pregnancy	3	1	2	2	3	
	ART	9	4	7	5	8	
NVS categories	Inadequate (0-1 points)	13	6	10	7	11	0.219
	Limited (2-3 points)	38	14	25	24	39	
	Adequate (4-6 points)	68	37	65	31	50	
SAHLSA categories	Inadequate (0-37 points)	17	6	10	11	18	0.261
	Adequate (38-50 points)	102	51	89	51	82	
SILS categories	Never	29	13	23	16	26	0.947
	Rarely	34	17	30	17	27	

For peer review only - http://bmjopen.bmj.com/site/about/guidelines.xhtml

	Sometimes	33	17	30	16	26	
	Often	8	4	7	4	6	
	Always	15	6	10	9	14	

[a]Chi-square; I: self-employed, higher professional or managerial employment; II: employee; III: student; IV: stay-at-home mother; V: unemployed; EU= European Union; ART= Assisted Reproduction Treatment.

The information and recommendation about vaccination came mainly from their midwives (94%), in 4% from the family doctor and 2% of women did not provide any information. As we wanted to be as sure as possible of the vaccination status of each participant, we validated the vaccination status reported by the participants with the immunization status recorded in the official electronic immunization registry. We corroborated that all women without immunisation recorded on the electronic record had not been vaccinated.

Regarding HL screening tools, the correlation between SAHLSA_50 and SILS was moderate, inversely proportional and significant (r= -0.251, p=0.007). The correlation between NVS and SAHLSA_50 was moderate and significant (r= 0.349, p<0.001). The correlation between NVS and SILS was moderate, inversely proportional and also significant (r= -0.307, p=0.001).

We also analysed the influence of participants' education on HL level and the scales of assessment. Higher education was directly related to higher SAHLSA_50 (r= 0.244, p<0.001) and NVS (r= 0.366, p=0.002) scores. This relationship however was not present in the SILS scale.

Vaccination status

17% (20/62) of women had been vaccinated against influenza prior to pregnancy. Gestational influenza vaccination coverage was 52% (62/119). The vaccine was administered to 5% (4/62) of women by week 20, and to 16% (10/62) in the last weeks of gestation (more than 36 weeks). Concerning pertussis vaccine, 94% (112/119) of women had it during pregnancy,

For peer review only - http://bmjopen.bmj.com/site/about/guidelines.xhtml

with 86% (96/112) vaccinated between weeks 27-32 of gestation. All women vaccinated against influenza were simultaneously vaccinated against whooping cough. There were no significant differences in sociodemographic or obstetric characteristics between pregnant vaccination status for influenza or pertussis (p=0.15 and p=0.35, respectively) [data not shown].

The reasons for rejection of women who were not vaccinated against influenza during pregnancy (57) are shown in **Figure 1**. 25% (14/57) felt that the vaccine was unnecessary, 23% (13/57) claimed to have received insufficient information from health professionals and 14% (8/57) claimed that they had never been infected. The reasons reported by women declining vaccination against pertussis were lack of information from health professionals (4/7 [57%]) and lack of any prenatal care (3/7 [43%]).

Figure 1 about here

Health literacy

In the NVS scale we obtained an average score of 3.7 ± 1.6 with values between 0 and 6. These scores were categorized as inadequate [13% (16/119)], limited [30% (36/119)] and adequate HL [56% (67)]. SAHLSA_50 scores were 44.1 ± 4.4 out of 50. An 86% (102/119) of women had adequate HL levels (SAHLSA-50 score>37). According to the SILS, 24% (29/119) women replied ‘never’ needing help when reading information, 29% (35/119) ‘rarely’, 27% (32/119) ‘sometimes’ and only 6% (7/119) replied ‘often’ and 13% (16/119) replied ‘always’.

To identify variables explaining HL levels according to each screening tool multivariate analyses were conducted. Multivariate lineal regression was used regarding the explanatory covariates for quantitative tools NVS and SALHSA_50. For these, the level of

For peer review only - http://bmjopen.bmj.com/site/about/guidelines.xhtml

education was found to be statistically significant (NVS [adjusted R-squared=0.258; p=0.001] and SALHSA_50 [adjusted R-squared=0.220; p=0.014]). A multinomial model was constructed for the qualitative scale SILS, observing no statistically significant differences.

There were no differences in NVS and SILS scores between women who declined and those who accepted influenza vaccination (Mann-Whitney U test, p=0.320 and p=0.942 respectively). However, for SAHLSA_50 (Median=44.5; IQR=5.0 *vs* 45.0; IQR=5.5) the differences were statistically significant (Mann-Whitney U test, p=0.019) (See **Figure 2**).

Figure 2 about here

Later, scores from the quantitative health literacy screening tools (NVS, SALHSA_50) were distributed in quartiles (**Figure 3**). For the NVS scale we found no statistically significant different between women who had accepted or declined vaccination (p=0.532). However, such difference was seen when using the SALHSA_50 tool (Kruskal-Wallis test, p=0.022). The median number of women vaccinated in the bottom quartile was 8 (95% CI: 7.0-9.0) versus 24 (95% CI: .23.0-25.0) in the top quartile.

We were interested in examining the characteristics of the women who were excluded from the study (49). We conducted an analysis of missing values for the three health literacy screening tools using the multiple imputation chained equations method.[31] Again, for the NVS scale we found no statistically significant difference between women who had accepted or declined vaccination (p=0.372) and, instead, such difference was seen when using the SALHSA_50 tool (Kruskal-Wallis test, p=0.003). The median number of women vaccinated in the bottom quartile was 11 (95% CI: 9.0-12.0) vs 28 (95% CI: 27.0-29.0) in the top quartile.

For peer review only - http://bmjopen.bmj.com/site/about/guidelines.xhtml

Figure 3 about here

Regarding the NVS, scores between pertussis-vaccinated and unvaccinated women were similar (Median= 4.0; IQR=0.0 *vs* Median=4.0; IQR=2.75), like the SAHLSA_50 scale (Median= 45.0; IQR=0.0 *vs* Median= 45.0; IQR=5.0). We also did not find any difference with the results from the SILS tool.

DISCUSSION

Vaccination is an essential public health intervention. We focused on pregnant women, an especially vulnerable population, and studied the acceptance of two vaccines underutilised in our community.[8] Few studies evaluating HL and vaccination have been conducted thus far and, up to now, none had focused on pregnant women.

In our study, influenza vaccination did not reach recommended levels although coverage slightly exceeded Australian[32] but not US rates.[33] In Valencia coverage has progressively improved from 2011 (8.5%) to 2015 (34.4%).[8] Regarding pertussis, the 97% vaccination rate improves on Belgian (39%)[34] or UK (70%)[6] rates. The disparity between pertussis and influenza immunization rates has not been previously addressed in detail.[6] We believe that in our setting, fear to pertussis –perhaps influenced by mass media[35] and fuelled by the increasing number of cases– could explain such high vaccination prevalence. Indeed, the pertussis vaccination programme was commenced following a surge in the number of cases and deaths. Clinicians may have therefore been keener to ensure that pregnant women got vaccinated and may have framed their advice more assertively. On the other hand, the disinterest from health professionals in providing information about influenza vaccination together with maternal perceptions that influenza vaccine was unnecessary were the most frequently cited causes of vaccine rejection, in agreement with prior studies.[5–8,36] This

For peer review only - http://bmjopen.bmj.com/site/about/guidelines.xhtml

position obviously ignores the benefits of acquired immunity for the newborn, which could reduce perinatal infections.[37]

We found that NVS classified 58% of participants with adequate HL. However, this figure increased up to 89% if SAHLSA_50 was used. Currently, there are no publications comparing both scales simultaneously in the same population. Such discrepancy between screening tools could be of much relevance as, of the tools pragmatically chosen for our research, only SALHSA_50 was predictive of vaccination in pregnant women. However, women with high SALHSA_50 scores were more likely to decline influenza vaccination, perhaps due to pre-conceived ideas; it might also be that women with high HL have more abilities to look for information on the internet or other sources and construct a narrative that supports such preconceptions, leading to decline this vaccination.[38] Such narratives would also no be challenged if professionals fail to adequately inform them or focus their persuasion solely on rational, data-based reasons instead of complementing such evidence with other emotional and behavioural aspects.[37,39,40] These results diverge from current evidence[4] in this group of women possibly highly involved in their health care, as already explored.[41]

As perhaps expected, HL screening results were directly related to the education of participants and thus, a higher level of education was associated with higher HL. Interestingly, other authors have reported that a higher level of education is associated with higher rates of vaccine rejection and hesitation.[36,42] In fact, it would appear that the emerging relationship between HL and vaccination described by those authors may be represented as an 'inverted U' shape' (i.e., high and low HL levels equally associated with low vaccination).

The analysis of missing values would help resolve some of the challenges originated from the incomplete responses. If cases with missing data were to be systematically different to cases with complete information, then results could be equivocal.[31] In our case, however,

For peer review only - http://bmjopen.bmj.com/site/about/guidelines.xhtml

the analysis of missing values did not produce different results to the original analysis conducted without imputed values.

Our study presents limitations. Although there are approximately 51 HL tools available,[17] experiences in Spain with these instruments have been few and limited to the Health Literacy Survey - European Union[43] or the eHealth Literacy (eHL)[44] tools. In addition, none of these tools have been validated in Spain, yet they have been so in Spanish-speaking US populations. Moreover, as there are no scales specifically focused on pregnant women, our questionnaire selection was eminently pragmatic and based on ease of use (SILS), robustness (SAHLSA_50) and reliability (NVS). Additionally, the routine use of HL screening tools in clinical practice remains nevertheless controversial, as such routine screening has shown no benefits yet could have undesirable effects for patients.[45]

Decisions related to vaccination may be influenced by the information provided, the communication approaches and attitudes of by health professionals.[36,37] Since there is currently no standardized approach to determine the abilities that pregnant women have to make effective use of the information provided, we hypothesise that information offered to each woman will be more or less similar and, therefore, women with low HL may be more likely to make suboptimal decisions because of such deficit. Logically, this does not consider efforts that professionals may make to compensate for any difficulties in understanding. Although exploring such efforts was outside the remit of our work, it would be interesting to investigate this aspect in future studies, together with any supporting materials used by professionals.

CONCLUSION

Vaccination is an essential public health measure and pregnant women can particularly benefit from this intervention. Identifying determinants of vaccination such as health literacy

For peer review only - http://bmjopen.bmj.com/site/about/guidelines.xhtml

would facilitate an adequate use of resources to encourage shared decision-making, ultimately resulting in optimal vaccination rates. Our findings suggesting a relation between high health literacy and rejection of vaccination encourage further research to identify and describe the factors involved in such relation and implement mitigating initiatives.

FUNDING

This work was supported by the Universidad Católica de Valencia "San Vicente Mártir" (Spain) through a competitive grant call [PRUCV/2015/639]. ECS is affiliated with the National Institute for Health Research (NIHR) Health Protection Research Unit (HPRU) in Healthcare Associated Infection and Antimicrobial Resistance at Imperial College London in partnership with Public Health England (PHE), and the NIHR Imperial Patient Safety Translational Research Centre. ECS has received an Early Career Research Fellowship from the Antimicrobial Research Collaborative at Imperial College London and acknowledges the support of the Florence Nightingale Foundation. The views expressed are those of the authors and not necessarily those of the NHS, the NIHR, the Department of Health, or Public Health England. The funders had no role in study design; collection, analysis, and interpretation of data; writing the report; and the decision to submit the report for publication.

For peer review only

COMPETING INTERESTS

None declared.

AUTHOR CONTRIBUTION

ECS RVC conceptualized and designed the study, RVC FSV collected data, ECS RVC FSV carried out the data analyses, ECS RVC ENI JDD FSV drafted the initial manuscript, ECS

For peer review only - http://bmjopen.bmj.com/site/about/guidelines.xhtml

RVC FSV ENI JDD reviewed and revised the manuscript. All authors read and approved the final manuscript.

DATA SHARING

No additional data are available.

ETHICAL APPROVAL

The study was conducted in compliance with the Declaration of Helsinki and it was approved by the research ethics and research committee of HULR on 10/07/15.

ACKNOWLEDGEMENTS

We are grateful to the Universidad Católica de Valencia and to the HULR for their support and participation in this project, and to all the women who have also taken part. We also thank Professor Daniel Lee for authorising the use of the SALHSA_50 tool in our research.

For peer review only

REFERENCES

1. Fiore AE, Uyeki TM, Broder K, et al. Prevention and control of influenza with vaccines: recommendations of the Advisory Committee on Immunization Practices (ACIP), 2010. *MMWR Recomm Rep*. 2010;59(RR-8):1-62. doi:10.1111/j.1600-6143.2011.03793.x.

2. Regan AK, Mak DB, Hauck YL, Gibbs R, Tracey L, Effler P V. Trends in seasonal influenza vaccine uptake during pregnancy in Western Australia: Implications for midwives. *Women and Birth*. 2016;29(5):423-429. doi:10.1016/j.wombi.2016.01.009.

3. Tamma PD, Ault KA, del Rio C, Steinhoff MC, Halsey NA, Omer SB. Safety of influenza vaccination during pregnancy. *Am J Obstet Gynecol*. 2009;201(6):547-552. http://www.ajog.org/article/S0002-9378(09)01108-9/pdf.

4. Yudin MH, Salaripour M, Sgro MD. Pregnant Women's Knowledge of Influenza and the Use and Safety of the Influenza Vaccine During Pregnancy. *J Obstet Gynaecol Canada*. 2009;31(2):120-125. doi:10.1016/S1701-2163(16)34095-6.

5. Panda B, Stiller R, Panda A. Influenza vaccination during pregnancy and factors for lacking compliance with current CDC guidelines. *J Matern Fetal Neonatal Med*. 2011;24(3):402-406. doi:10.3109/14767058.2010.497882.

6. Laenen J, Roelants M, Devlieger R, Vandermeulen C. Influenza and pertussis vaccination coverage in pregnant women. *Vaccine*. 2015;33(18):2125-2131.

For peer review only - http://bmjopen.bmj.com/site/about/guidelines.xhtml

doi:10.1016/j.vaccine.2015.03.020.

7. Ding H, Black CL, Ball S, et al. Influenza Vaccination Coverage Among Pregnant Women--United States, 2014-15 Influenza Season. *MMWR Morb Mortal Wkly Rep*. 2015;64(36):1000-1005. doi:10.15585/mmwr.mm6436a2.

8. Vila-Candel R, Navarro-Illana P, Navarro-Illana E, et al. Determinants of seasonal influenza vaccination in pregnant women in Valencia, Spain. *BMC Public Health*. 2016;16:1173. doi:10.1186/s12889-016-3823-1.

9. Torres J, Godoy P, Artigues A, et al. [Outbreak of whooping cough with a high attack rate in well-vaccinated children and adolescents]. *Enfermedades Infecc y Microbiol clínica*. 2011;29(8):564-567. doi:10.1016/j.eimc.2011.04.005.

10. Amirthalingam G, Andrews N, Campbell H, et al. Effectiveness of maternal pertussis vaccination in England: An observational study. *Lancet*. 2014;384(9953):1521-1528. doi:10.1016/S0140-6736(14)60686-3.

11. WHO. Pertussis vaccines: WHO position paper, August 2015-Recommendations. *Vaccine*. 2016;34(12):1423-1425. doi:10.1016/j.vaccine.2015.10.136.

12. Situación de la Tos ferina en España, 2005-2016. Centro Nacional de Epidemiología. Minesterio de Economía, Industria y Competitividad. http://www.isciii.es/ISCIII/es/contenidos/fd-servicios-cientifico-tecnicos/fd-vigilancias-alertas/fd-enfermedades/pdf_2016/INFORME_Tos_ferina_Espana_2005-2016.pdf. Published 2016. Accessed October 20, 2017.

13. Sørensen K, Van den Broucke S, Fullam J, et al. Health literacy and public health: A systematic review and integration of definitions and models. *BMC Public Health*. 2012;12(1):80. doi:10.1186/1471-2458-12-80.

14. Baker DW. The meaning and the measure of health literacy. *J Gen Intern Med*. 2006;21(8):878-883. doi:10.1111/j.1525-1497.2006.00540.x.

For peer review only - http://bmjopen.bmj.com/site/about/guidelines.xhtml

15. Nutbeam D. Health literacy as a public goal: a challenge for contemporary health education and communication strategies into the 21st century. *Heal Promot Int*. 2000;15(3):259-267. doi:10.1093/heapro/15.3.259.

16. Heijmans M, Uiters E, Rose T, et al. *Study on Sound Evidence for a Better Understanding of Health Literacy in the European Union*.; 2015. doi:10.2818/150402.

17. Haun JN, Valerio MA, McCormack LA, Sørensen K, Paasche-Orlow MK. Health literacy measurement: An inventory and descriptive summary of 51 instruments. *J Health Commun*. 2014;19:302-333. doi:10.1080/10810730.2014.936571.

18. Sørensen K, Pelikan JM, Röthlin F, et al. Health literacy in Europe: comparative results of the European health literacy survey (HLS-EU). *Eur J Public Health*. 2015;25(6):1053-1058. doi:10.1093/eurpub/ckv043.

19. Lee S-YD, Stucky BD, Lee JY, Rozier RG, Bender DE. Short Assessment of Health Literacy-Spanish and English: a comparable test of health literacy for Spanish and English speakers. *Health Serv Res*. 2010;45(4):1105-1120. doi:10.1111/j.1475-6773.2010.01119.x.

20. Vilca Yengle LM, Campins Martí M, Cabero Roura L, et al. [Influenza vaccination in pregnant women. Coverage, practices and knowledge among obstetricians]. *Med Clin (Barc)*. 2010;134(4):146-151.

21. Castro-Sánchez E, Chang PWS, Vila-Candel R, Escobedo AA, Holmes AH. Health literacy and infectious diseases: why does it matter? *Int J Infect Dis*. 2016;43:103-110. doi:10.1016/j.ijid.2015.12.019.

22. Morrison AK, Schapira MM, Hoffmann RG, Brousseau DC. Measuring health literacy in caregivers of children: a comparison of the newest vital sign and S-TOFHLA. *Clin Pediatr (Phila)*. 2014;53(13):1264-1270. doi:10.1177/0009922814541674.

23. Warsh J, Chari R, Badaczewski A, Hossain J, Sharif I. Can the Newest Vital Sign be

For peer review only - http://bmjopen.bmj.com/site/about/guidelines.xhtml

used to assess health literacy in children and adolescents? *Clin Pediatr (Phila)*. 2014;53(2):141-144. doi:10.1177/0009922813504025.

24. Weiss BD, Mays MZ, Martz W, et al. Quick assessment of literacy in primary care: The newest vital sign. *Ann Fam Med*. 2005;3(6):514-522. doi:10.1370/afm.405.

25. Morris NS, MacLean CD, Chew LD, Littenberg B. The Single Item Literacy Screener: evaluation of a brief instrument to identify limited reading ability. *BMC Fam Pract*. 2006;7:21. doi:10.1186/1471-2296-7-21.

26. Mak TK, Mangtani P, Leese J, Watson JM, Pfeifer D. Influenza vaccination in pregnancy: current evidence and selected national policies. *Lancet Infect Dis*. 2008;8(1):44-52.

27. Davis RE, Couper MP, Janz NK, Caldwell CH, Resnicow K. Interviewer effects in public health surveys. *Health Educ Res*. 2010;25(1):14-26. http://dx.doi.org/10.1093/her/cyp046.

28. Padilla-Santoyo P, Vílchez-Román C. Psychometric properties of the SAHLSA-50, a standardized test to evaluate the health literacy. *Rev Per Obs Enf*. 2008;4(2):90-95.

29. Delanoë A, Lépine J, Leiva Portocarrero ME, et al. Health literacy in pregnant women facing prenatal screening may explain their intention to use a patient decision aid: a short report. *BMC Res Notes*. 2016;9(1):339. doi:10.1186/s13104-016-2141-0.

30. Vila-Candel R, Navarro-Illana P, Navarro-Illana E, et al. Determinants of seasonal influenza vaccination in pregnant women in Valencia, Spain. *BMC Public Health*. 2016;16(1):1173. doi:10.1186/s12889-016-3823-1.

31. Hayati Rezvan P, Lee KJ, Simpson JA. The rise of multiple imputation: a review of the reporting and implementation of the method in medical research. *BMC Med Res Methodol*. 2015;15(1):30. doi:10.1186/s12874-015-0022-1.

32. Maher L, Hope K, Torvaldsen S, et al. Influenza vaccination during pregnancy:

For peer review only - http://bmjopen.bmj.com/site/about/guidelines.xhtml

Coverage rates and influencing factors in two urban districts in Sydney. *Vaccine*. 2013;31(47):5557-5564. doi:10.1016/j.vaccine.2013.08.081.

33. Henninger M. Trends in Influenza Vaccine Coverage in Pregnant Women, 2008 to 2012. *Perm J*. 2013;17(2):31-36. doi:10.7812/TPP/12-115.

34. Maertens K, Caboré RN, Huygen K, Hens N, Van Damme P, Leuridan E. Pertussis vaccination during pregnancy in Belgium: Results of a prospective controlled cohort study. *Vaccine*. 2016;34(1):142-150. doi:10.1016/j.vaccine.2015.10.100.

35. Campins M, Moreno-Perez D, Gil-de Miguel A, et al. [Whooping cough in Spain. Current epidemiology, prevention and control strategies. Recommendations by the Pertussis Working Group]. *Enferm Infecc Microbiol Clin*. 2013;31(4):240-253. doi:10.1016/j.eimc.2012.12.011.

36. Biasio LR. Vaccine hesitancy and health literacy. *Hum Vaccin Immunother*. 2017;13(3):701-702. doi:10.1080/21645515.2016.1243633.

37. Lorini C, Santomauro F, Donzellini M, et al. Health literacy and vaccination: A systematic review. *Hum Vaccin Immunother*. October 2017:00-00. doi:10.1080/21645515.2017.1392423.

38. Stahl JP, Cohen R, Denis F, et al. The impact of the web and social networks on vaccination. New challenges and opportunities offered to fight against vaccine hesitancy. *Med Mal Infect*. 2016;46(3):117-122. doi:10.1016/j.medmal.2016.02.002.

39. Niccolai LM, Pettigrew MM. The Role of Cognitive Bias in Suboptimal HPV Vaccine Uptake. *Pediatrics*. 2016;138(4):e20161537.

40. Yaqub O, Castle-Clarke S, Sevdalis N, Chataway J. Attitudes to vaccination: A critical review. *Soc Sci Med*. 2014;112:1-11. doi:10.1016/j.socscimed.2014.04.018.

41. Fadda M, Depping MK, Schulz PJ. Addressing issues of vaccination literacy and psychological empowerment in the measles-mumps-rubella (MMR) vaccination

For peer review only - http://bmjopen.bmj.com/site/about/guidelines.xhtml

decision-making: a qualitative study. *BMC Public Health*. 2015;15:836. doi:10.1186/s12889-015-2200-9.

42. Hak E, Schnbeck Y, De Melker H, Van Essen GA, Sanders EAM. Negative attitude of highly educated parents and health care workers towards future vaccinations in the Dutch childhood vaccination program. *Vaccine*. 2005;23(24):3103-3107. doi:10.1016/j.vaccine.2005.01.074.

43. Sorensen K, Van den Broucke S, Pelikan JM, et al. Measuring health literacy in populations: illuminating the design and development process of the European Health Literacy Survey Questionnair (HLS-EU-Q). *BMC Public Health*. 2013;13(1):948. doi:10.1186/1471-2458-13-948.

44. Paramio Perez G, Almagro BJ, Hernando Gomez A, Aguaded Gomez JI. [Validation of the eHealth Literacy Scale (eHEALS) in Spanish University Students]. *Rev Esp Salud Publica*. 2015;89(3):329-338. doi:10.4321/s1135-57272015000300010.

45. Dickens C. Health Literacy and Nursing. *AJN, Am J Nurs*. 2013;113(6):52-57. doi:10.1097/01.NAJ.0000431271.83277.2f.

For peer review only

For peer review only - http://bmjopen.bmj.com/site/about/guidelines.xhtml

FIGURE TITLES

Figure 1. Reasons given by participants to decline influenza vaccination

Figure 2. Relationship between acceptance of influenza vaccination and SAHLSA_50 scale (N=119)

Figure 3. Relationship between acceptance of influenza vaccination and SAHLSA_50 scale distribution by quartiles (N=119)

For peer review only

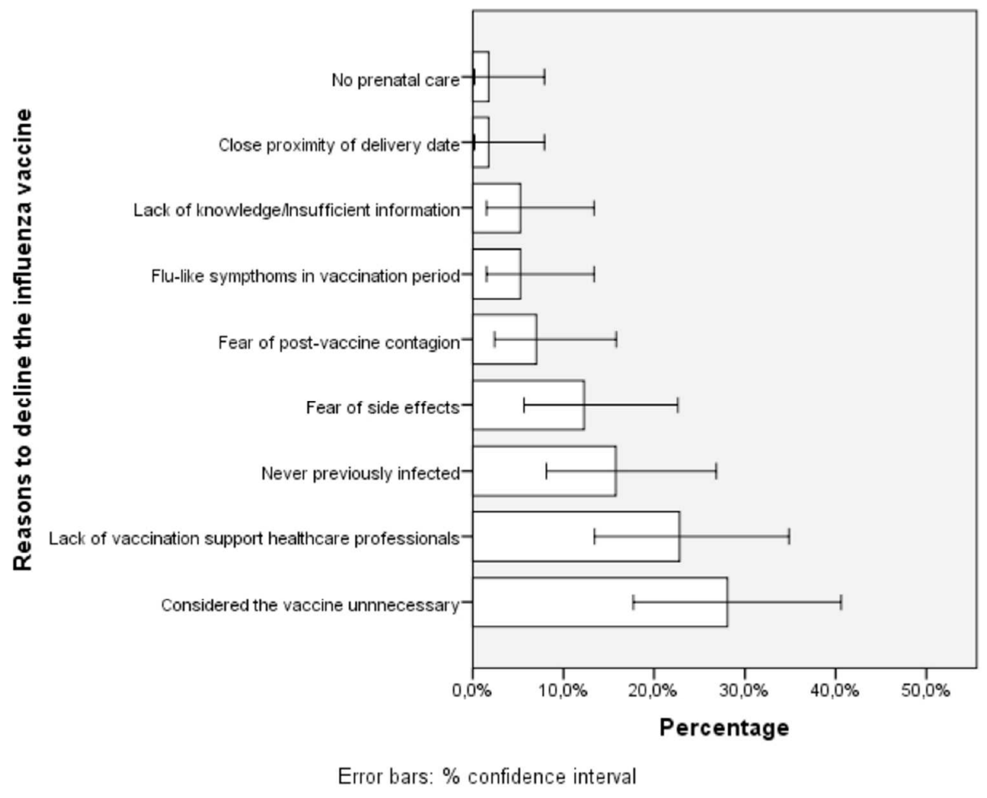

Figure 1. Reasons given by participants to decline influenza vaccination

79x63mm (300 x 300 DPI)

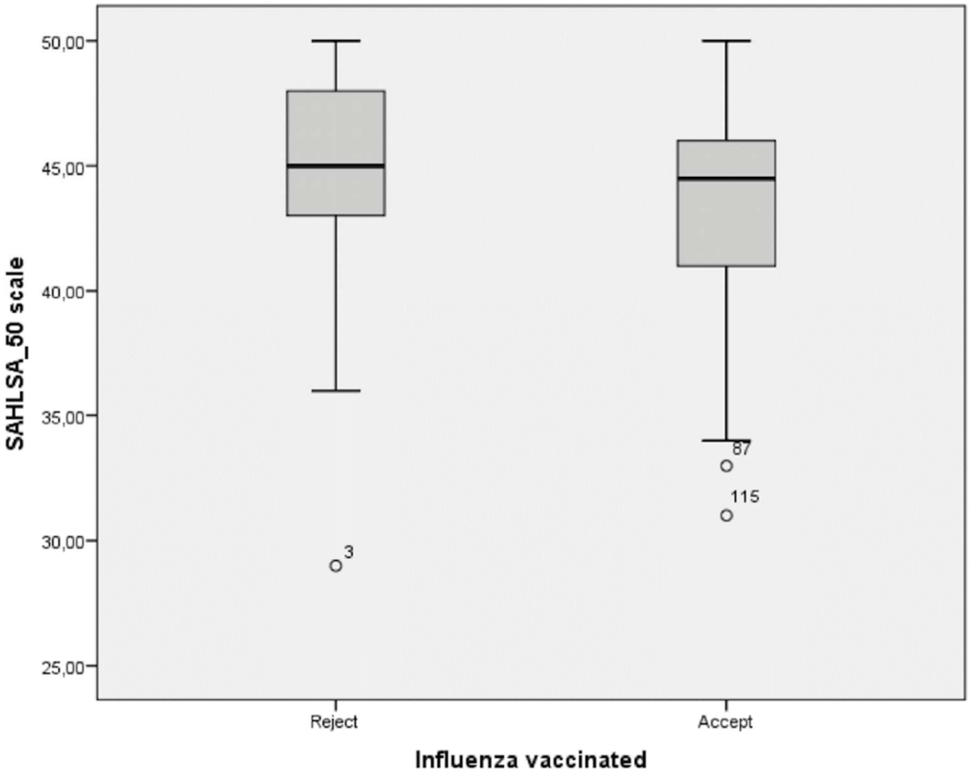

Figure 2. Relationship between acceptance of influenza vaccination and SAHLSA_50 scale (N=119)

81x65mm (300 x 300 DPI)

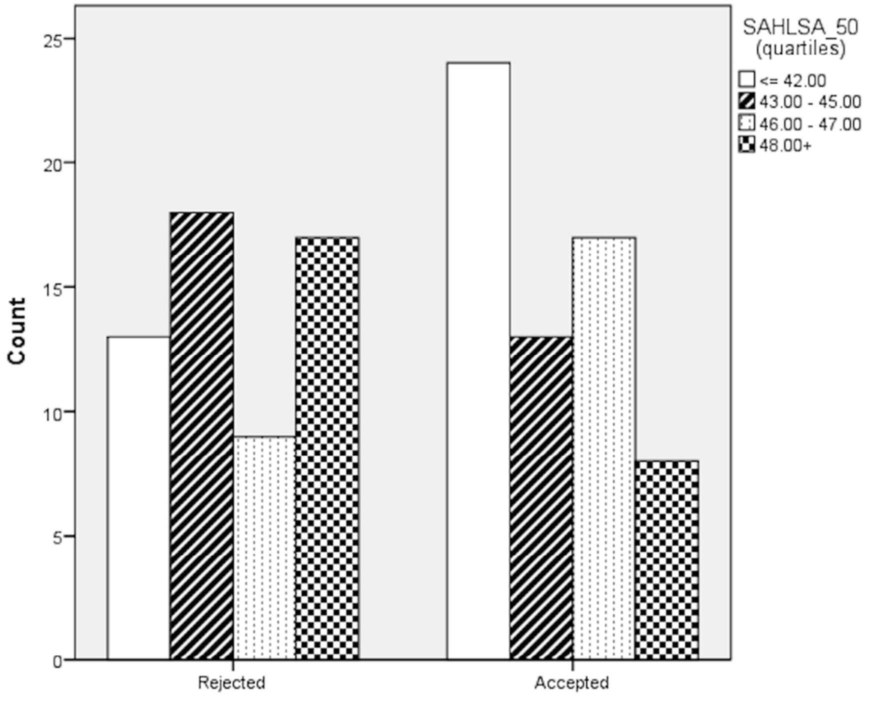

Figure 3. Relationship between acceptance of influenza vaccination and SAHLSA_50 scale distribution by quartiles (N=119)

81x65mm (300 x 300 DPI)

STROBE 2007 (v4) Statement—Checklist of items that should be included in reports of *cross-sectional studies*

Section/Topic	Item #	Recommendation	Reported on page #
**Title and abstract**	1	(*a*) Indicate the study's design with a commonly used term in the title or the abstract	1
		(*b*) Provide in the abstract an informative and balanced summary of what was done and what was found	2
**Introduction**			
Background/rationale	2	Explain the scientific background and rationale for the investigation being reported	5,6
Objectives	3	State specific objectives, including any prespecified hypotheses	5
**Methods**			
Study design	4	Present key elements of study design early in the paper	6
Setting	5	Describe the setting, locations, and relevant dates, including periods of recruitment, exposure, follow-up, and data collection	6,7
Participants	6	(*a*) Give the eligibility criteria, and the sources and methods of selection of participants	6
Variables	7	Clearly define all outcomes, exposures, predictors, potential confounders, and effect modifiers. Give diagnostic criteria, if applicable	7,8
Data sources/ measurement	8*	For each variable of interest, give sources of data and details of methods of assessment (measurement). Describe comparability of assessment methods if there is more than one group	7,8
Bias	9	Describe any efforts to address potential sources of bias	7,8
Study size	10	Explain how the study size was arrived at	7
Quantitative variables	11	Explain how quantitative variables were handled in the analyses. If applicable, describe which groupings were chosen and why	8,9
Statistical methods	12	(*a*) Describe all statistical methods, including those used to control for confounding	8,9
		(*b*) Describe any methods used to examine subgroups and interactions	8,9
		(*c*) Explain how missing data were addressed	13
		(*d*) If applicable, describe analytical methods taking account of sampling strategy	
		(*e*) Describe any sensitivity analyses	
**Results**			

For peer review only

Participants	13*	(a) Report numbers of individuals at each stage of study—eg numbers potentially eligible, examined for eligibility, confirmed eligible, included in the study, completing follow-up, and analysed	9
		(b) Give reasons for non-participation at each stage	9
		(c) Consider use of a flow diagram	
Descriptive data	14*	(a) Give characteristics of study participants (eg demographic, clinical, social) and information on exposures and potential confounders	9,10
		(b) Indicate number of participants with missing data for each variable of interest	11
Outcome data	15*	Report numbers of outcome events or summary measures	10-13
Main results	16	(*a*) Give unadjusted estimates and, if applicable, confounder-adjusted estimates and their precision (eg, 95% confidence interval). Make clear which confounders were adjusted for and why they were included	10-13
		(*b*) Report category boundaries when continuous variables were categorized	
		(*c*) If relevant, consider translating estimates of relative risk into absolute risk for a meaningful time period	
Other analyses	17	Report other analyses done—eg analyses of subgroups and interactions, and sensitivity analyses	
**Discussion**			
Key results	18	Summarise key results with reference to study objectives	14
Limitations	19	Discuss limitations of the study, taking into account sources of potential bias or imprecision. Discuss both direction and magnitude of any potential bias	15
Interpretation	20	Give a cautious overall interpretation of results considering objectives, limitations, multiplicity of analyses, results from similar studies, and other relevant evidence	14,15
Generalisability	21	Discuss the generalisability (external validity) of the study results	16
**Other information**			
Funding	22	Give the source of funding and the role of the funders for the present study and, if applicable, for the original study on which the present article is based	17

*Give information separately for cases and controls in case-control studies and, if applicable, for exposed and unexposed groups in cohort and cross-sectional studies.

Note: An Explanation and Elaboration article discusses each checklist item and gives methodological background and published examples of transparent reporting. The STROBE checklist is best used in conjunction with this article (freely available on the Web sites of PLoS Medicine at http://www.plosmedicine.org/, Annals of Internal Medicine at http://www.annals.org/, and Epidemiology at http://www.epidem.com/). Information on the STROBE Initiative is available at www.strobe-statement.org.

For peer review only
